# Imidazoline-I2 PET Tracers in Neuroimaging

**DOI:** 10.3390/ijms24129787

**Published:** 2023-06-06

**Authors:** Christine A. Parker, David J. Nutt, Robin J. Tyacke

**Affiliations:** 1 Neuropsychopharmacology Unit, Division of Psychiatry, Imperial College London, Hammersmith Hospital Campus, Du Cane Road, London W12 0NN, UK; 2 GlaxoSmithKline, Gunnels Wood Road, Stevenage SG1 2NY, UK

**Keywords:** imidazoline binding sites, I_2_BS, PET, astrogliosis

## Abstract

Targeting neuroinflammation, and in particular, microglial activation and astrocytosis, is a current area of the focus of new treatment interventions for a number of neurodegenerative disorders. Probing the roles of microglia and astrocytes in human disease requires the development of useful tools, such as PET imaging tools that are specific for the cell type(s) of interest. This review concentrates on the recent advances in the development of Imidazoline_2_ binding site (I_2_BS) PET tracers, which are purported to target astrocytes, and hence could represent key clinical imaging tools for targeting astrocytes in neurodegenerative disease. Five PET tracers for the I_2_BS are described in this review, with only one (^11^C-BU99008) being currently validated to GMP for clinical use, and data reported from healthy volunteers, Alzheimer’s disease patients, and Parkinson’s disease patients. The clinical data utilising ^11^C-BU99008 have revealed the potential early involvement of astrogliosis in neurodegeneration that might precede the activation of microglia, which, if confirmed, could provide a vital new means for potentially targeting neurodegeneration earlier in the disease course.

## 1. Introduction

Imidazoline receptors or binding sites were discovered from studies using the α_2_-adrenoceptor agonist clonidine and the antagonist idazoxan. Both drugs have actions and bind to the discrete populations of binding sites that are not attributable to the α_2_-adrenoceptor. This non-α_2_-adrenoceptor phenomenon was originally defined as Imidazoline receptors/binding sites. Those that preferentially bound clonidine were named Imidazoline-1 (I_1_) and those that preferentially bound idazoxan were named Imidazoline-2 (I_2_). The detailed pharmacology, function, and background of these Imidazoline receptors/binding sites are out of the scope of this review, but there have been many recent reviews detailing them [1].

The development of a successful PET tracer follows many of the same steps as those required for any small molecule that is intended for use in vivo, e.g., the affinity and specificity of its biological target and the appropriate safety profile, etc. In fact, many good PET tracers are already repurposed drugs, such as ^11^C-carfentanil and ^11^C-raclopride. However, in addition, a successful PET tracer must also satisfy other specific criteria, as detailed below:

### Ideal Criteria for Good PET Tracer

- An amenability to labelling
- A high selectivity
- A high affinity
- Minimal lipophilicity
- Good blood–brain barrier (BBB) penetration (for CNS tracers)
- Insignificant or a lack of problematic metabolism
- Acceptable pharmacokinetics
- A lack of pharmacological effect at doses used
- Acceptable safety
- A good test–re-test reliability

Meeting all of these criteria is difficult and many potential PET tracers fail to do so, meaning they do not progress to being used in either preclinical PET or clinical PET. There are many PET ligands that do meet enough criteria to be used and, in the criteria that they do not meet “ideally”, they either meet sufficiently well to be useable, or these “less” than ideal criteria are accounted for though experimental design.

**Figure 1:**
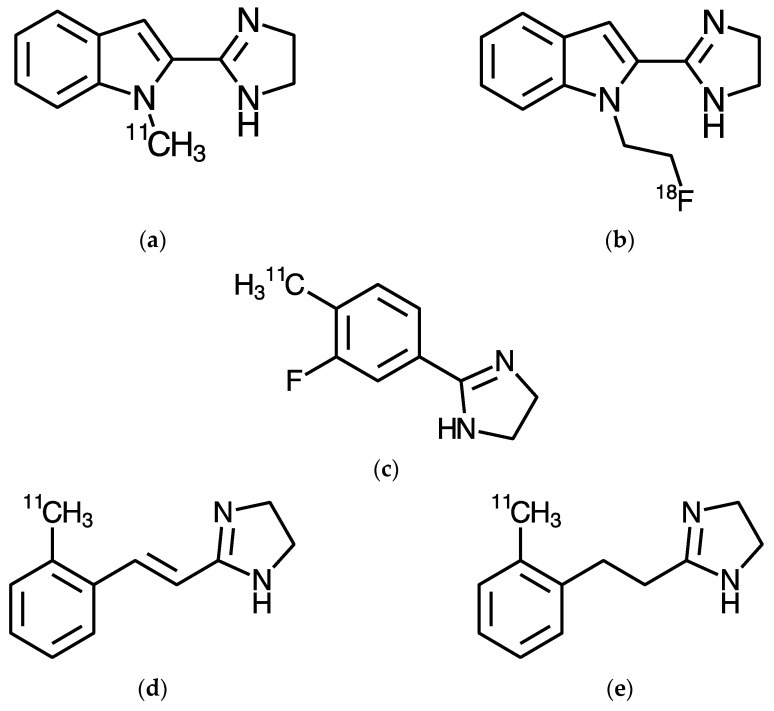
Structures of the current PET tracers for the I_2_BS: (**a**) ^11^C-BU99008; (**b**) ^18^F-FEBU (^18^F-BU99018); (**c**) ^11^C-FTIMD; (**d**) ^11^C-Metrazoline; and (**e**) ^11^C-TEIMD.

In this review, we look specifically at the development and utilisation of PET tracers for the Imidazoline_2_ Binding Site (I_2_BS). There have been a range of I_2_BS PET tracers developed (Figure 1) [2,3,4,5]. Some of these have been evaluated in preclinical species [2,3,4,5,6,7,8], but to date, only one I_2_BS tracer, ^11^C-BU99008, has been evaluated in humans, both in healthy subjects [9,10] and patient populations [11,12,13,14]. While the I_2_BS has been implicated in a range of different biological processes, the possibility that it is a marker for astrocytes has been of the most interest. It is this that has driven much of the development and subsequent use of the tracers for the I_2_BS discussed in this review.

## 2. I_2_ PET Tracers

To date, there have been five PET tracers developed for the I_2_BS: ^11^C-BU99008, ^18^F-FEBU (^18^F-BU99018), ^11^C-FTIMD, ^11^C-Metrazoline, and ^11^C-TEIMD, which are discussed in more detail below. Figure 1 shows the structures of these tracers and Table 1 summarises their key features and the species in which they have been evaluated and used.

### 2.1. ^11^C-BU99008

#### 2.1.1. Radiochemistry

**Figure 2:**
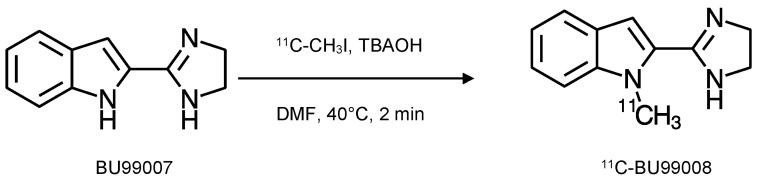
^11^C-BU99008 synthesis with N-alkylation of the precursor BU99007 using ^11^C-CH_3_I. BU99007 was dissolved in dimethylformamide and mixed with tetrabutylammonium hydroxide (0.1 M methanol solution). ^11^C-CH_3_I was delivered and the mixture heated to 40 °C for 2 min. Semi-preparative HPLC was used to purify the ^11^C-BU99008, followed by C18 Sep-Pak (Waters), rinsed with water (10 mL), and eluted off with ethanol (2 mL), followed by saline (8 mL) (adapted from [9]).

BU99008 (Figure 1) was initially synthesised as part of a series of potential PET tracers that could be easily radiolabelled from the same precursor, BU99007 [15]. Radiolabelling with carbon-11 was achieved via the alkylation of BU99007 using ^11^C-methyl iodide, providing a good yield and specific activity (76 ± 27 GBq·µmol^−1^) [5,19]. The exact conditions of this alkylation reaction have been altered since the initial porcine study [5,19] via a study in non-human primates (146 ± 33 GBq·µmol^−1^) [8], with the optimum conditions now implemented for the GMP production of ^11^C-BU99008 for human use (51 ± 20 GBq·µmol^−1^) [9] (Figure 2).

While ^11^C-BU99008 has proved to be a good tracer for imaging the I_2_BS in several species, including humans [9], NHPs [8], and porcine [5], its nano-molar affinity for the I_2_BS [15] makes it a complex ligand for use in smaller species, due to the injected mass potentially causing a self-block with the carbon-11 version of BU99008. To overcome this, an ultra-high specific activity (SA) synthesis for ^11^C-BU99008 was developed [7]. This method successfully produced an SA from the ultra-high synthesis of 60–120 MBq/6.6–12 pmol (9000 to 10,000 GBq·µmol^−1^), in comparison with a SA for the conventional synthesis of 77–140 MBq/0.50–1.1 nmol (127 to 154 GBq·µmol^−1^). This ultra-high synthesis method resulted in a superior specific binding signal for ^11^C-BU99008 to the I_2_BS and other indicators of specific radioactive accumulation [7].

#### 2.1.2. In Vitro Pharmacology

BU99008 has been shown to be a good compound for the I_2_BS, with a high affinity and selectivity [15]. Equilibrium binding constants derived using three different techniques were all in good agreement, showing BU99008 to have a nano-molar affinity for the I_2_BS. Kinetic binding enabled the calculation of the equilibrium association and dissociation constants of K_a_ = 0.88 ± 0.23 nM^−1^ and K_d_ = 1.2 ± 0.3 nM, respectively. The displacement of ^3^H-2BFI, another high-affinity I_2_BS-specific ligand, by BU99008, gave a K_i_ = 1.4 ± 0.6 nM and the saturation of ^3^H-BU99008, resulting in a K_d_ = 1.3 ± 0.5 nM. In addition to the affinity of BU99008 for the I_2_BS, its selectivity for the α_2_-adrenoceptor (1273 nM) was also determined and found to be 909-fold lower for the α_2_-adrenoceptor vs. the I_2_BS. The displacement binding of ^3^H-BU99008 via a range of other known I_2_BS compounds and non-I_2_BS compounds showed it to have a pharmacological profile very similar to other known high-affinity and selective I_2_BS compounds, such as 2BFI and BU224 [20]. Ex vivo biodistribution studies using ^3^H-BU99008 in rats were able to show that ^3^H-BU99008 had a good brain uptake following peripheral administration. This brain uptake was shown to be heterogenous and consistent with the known distribution of the I_2_BS. Furthermore, this uptake was blocked by prior treatment with BU224 (6 mg·kg^−1^, i.p.), and importantly, reduced to uniform levels throughout the brain areas measured [15].

#### 2.1.3. In Vivo Preclinical

The ability of ^11^C-BU99008 to label the I_2_BS has been validated in vivo in several preclinical species, including porcine [5] and NHPs [8]. In porcine, it was found that ^11^C-BU99008 showed a high brain uptake, with the highest uptake occurring in the thalamus and the lowest in the cortex and cerebellum. The kinetics of this uptake were best described by a one-tissue-compartment model (1TCM). This enabled the volume of distribution (*V*_T_) across the brain regions of interest to be calculated, showing a heterogeneous distribution: thalamus > striatum > hippocampus > frontal cortex ≥ cerebellum. This distribution was consistent with the known distribution and concentration of the I_2_BS. In addition, it was demonstrated that the uptake could be blocked in a dose-dependent manner via prior administration of the I_2_BS compound BU224, with the highest dose (5 mg·kg^−1^) reducing the V_T_ to homogeneous levels in all brain regions [5].

There were similar findings when ^11^C-BU99008 was assessed in NHPs. Again, there was a high brain uptake that showed a heterogenous distribution, consistent with that of the I_2_BS in NHP brains. This distribution: globus pallidus > cortical regions > cerebellum, was consistent with the reported regional I_2_BS densities, as determined by autoradiography in human tissue sections and the preclinical in vivo PET studies on porcine mentioned above. The kinetics of the in vivo administration of ^11^C-BU99008 were reversible and specific, but were not described well by a 1TCM, as was determined in the porcine study. However, the two-tissue-compartment model (2TCM) did describe the in vivo kinetics better for the NHPs, but as with the 1TCM, it was not ideal. A further analysis determined that the best model to fit for the in vivo NHP data was the multilinear analysis (MA1) model. It was also shown that this uptake could be blocked by prior administration of BU224 (0.01–0.3 mg·kg^−1^), with a whole brain ED_50_ = 0.022 mg·kg^−1^ in a saturable, dose-dependent manner. Moreover, pre-treatment with BU224 decreased the ^11^C-BU99008 binding in all regions of the brain assessed, further demonstrating the heterogeneous distribution of I_2_BS proteins in NHP brains and the binding specificity for this radiotracer [8].

Previously, some monoamine oxidase (MAO) enzymes inhibitors have been shown to affect the binding of the I_2_BS, as well as other I_2_BS-related signals, both in vitro and in vivo, which was reviewed in [1]. This has led to the assumption that the I_2_BS and MAO enzymes are in some way related. However, pre-treatment with either an MAO_A_ inhibitor (moclobemide) or MAO_B_ inhibitor (lazabemide) did not result in any significant change in the binding signal of ^11^C-BU99008 to any regions studied in the NHP brains, further demonstrating the specificity of ^11^C-BU99008 to the I_2_BS [8].

Both these reports of ^11^C-BU99008 in large preclinical species [5,8] indicated that this tracer would represent a good PET tracer for assessing the I_2_BS in humans (see section below; [9]). As mentioned above, imaging the I_2_BS with ^11^C-BU99008 was found to be less useful in smaller preclinical species, where its high affinity led to a poor signal due to self-block. The development of a method for producing an ultra-high SA (UHSA)-labelled version of ^11^C-BU99008, as mentioned above, has now overcome this limitation, and provides a means for better understanding the role of the I_2_BS in rodent models of disease, as it provides a significantly better signal and enables a clear delineation of brain regions in small animal PET in comparison with the conventional SA method [7]. The UHSA ^11^C-BU99008 has now shown significantly higher levels of the tracer to be taken up and retained in the hypothalamus and hippocampus of Zucker fatty rats compared with Zucker lean rats [21]; hence, this also demonstrates the utility of this UHSA form of ^11^C-BU99008 in understanding the role of I_2_BS non-CNS disease models using small animal PETs.

#### 2.1.4. Dosimetry

A dosimetry study was conducted with ^11^C-BU99008 in male humans, showing that there were no unexpected or dangerous accumulations of this radiotracer in any tissues. The highest mean absorbed dose was in the heart wall (0.028 ± 0.002 mGy·MBq^−1^). The total mean effective dose averaged over the subjects was estimated to be 0.0056 ± 0.0004 mSv·MBq^−1^. This resulted in a total effective dose of 1.96 mSv for a typical injection of 350 MBq of ^11^C-BU99008, which is not appreciably different from those obtained with other carbon-11 tracers [10].

### 2.2. ^18^F-FEBU (^18^F-BU99018)

#### 2.2.1. Radiochemistry

**Figure 3:**
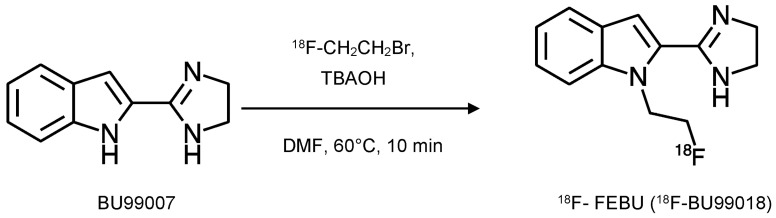
^18^F-FEBU synthesis via N-alkylation of the precursor BU99007 using ^18^F-CH_2_CH_2_Br. BU99007 was mixed with 1 mol/L tetrabutylammonium hydroxide (TBAOH) in methanol in anhydrous N,N-dimethylformamide (DMF) at room temperature, and heated to 60 °C for 10 min. Purification was via HPLC and fractions of ^18^F-FEBU were collected, dried, and redissolved in physiological saline for use (adapted from [4]).

FEBU (BU99018) (Figure 1 and Figure 3) was initially synthesised as part of the same series of potential PET tracers as BU99008 [15]. It is the fluoroethyl derivative, while BU99008 is the methyl derivative. Radiolabelling with fluorine-18 was achieved as described in [4]. This was a fluoro-ethylation of the precursor, BU99007, with ^18^F-fluoroethyl bromide in the presence of tetrabutylammonium hydroxide (TBAOH) (Figure 3). The successful synthesis of ^18^F-FEBU produced a decay-corrected radiochemical yield of 10.1 ± 5.3%, a specific activity of 40–147 GBq·µmol^−1^, and a radiochemical purity of >99 % at the end of the synthesis.

#### 2.2.2. In Vitro Pharmacology

FEBU (BU99018) was part of a series of potential I_2_BS PET tracers first described in [15,22]. It showed a nano-molar affinity for the I_2_BS, K_i_ = 2.6 ± 0.9 nM, and a good selectivity in comparison with the α_2_-adrenoceptor (870-fold). FEBU is the fluoroethyl derivative of the series. Originally, it was thought to be problematic due to its poor stability in solutions, particularly MeOH, where a slow conversion to a cyclised by-product at room temperature occurred [15]. However, this does not seem to be a major problem over the time scale required for its short-term use as a PET tracer [4]. This fluoroethyl derivative was developed to enable the use of the longer half-life positron emitter 18-flourine. The rationale is that this would allow its increased use across PET centres by permitting “off-site” synthesis and transfer across sites, but the instability of FEBU may become more of an issue in such situations, and so requires evaluation.

#### 2.2.3. In Vivo Preclinical

The utility of ^18^F-FEBU as a PET tracer for the I_2_BS was evaluated in vivo in mice and rats [4]. In mice, ^18^F-FEBU showed a rapid uptake in both peripheral and brain regions, which decreased over time. Pre-treatment with the I_2_BS ligands BU224 and 2BFI (1 mg·kg^−1^) significantly blocked this uptake in the brain and some other tissues. The uptake in rat brains was also found to be rapid and heterogeneous, with a high radioactivity uptake observed in the hypothalamus (including the arcuate nuclei) and hippocampus, and a moderate radioactivity uptake observed in the cerebellum. As it did in the mice, pre-treatment with the I_2_BS ligand BU224 (1 mg·kg^−1^) significantly blocked uptake in all the regions studied.

### 2.3. ^11^C-FTIMD 

#### 2.3.1. Radiochemistry

**Figure 4:**
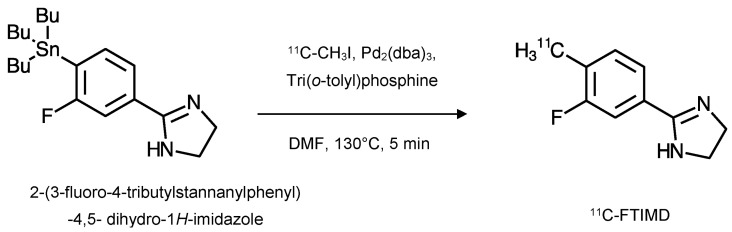
^11^C-FTIMD was synthesised from the tributylstannyl precursor (2-(3-fluoro-4-tributylstannanylphenyl)-4,5- dihydro-1*H*-imidazole) using ^11^C-methyl iodide. The precursor was mixed with a solution of tris(dibenzylideneacetone)dipalladium(0) (Pd_2_(dba)_3_; 1 μmol) and tri(*o*-tolyl)phosphine (4 μmol) in anhydrous *N*,*N*-dimethylformamide (DMF). The reaction mixture was heated to 130 °C for 5 min. Purification was via HPLC and fractions of ^11^C-FTIMD were collected, dried, and redissolved in physiological saline for use (adapted from [3]).

^11^C-FTIMD was initially synthesised (Figure 4) via a palladium-promoted cross-coupling reaction, but in a somewhat low radiochemical yield (5.4 ± 2.0%; decay-corrected). Its specific activity was 108 ± 33 GBq·µmol^−1^ at the end of this synthesis and it had a radiochemical purity of >95% [3].

**Figure 5:**
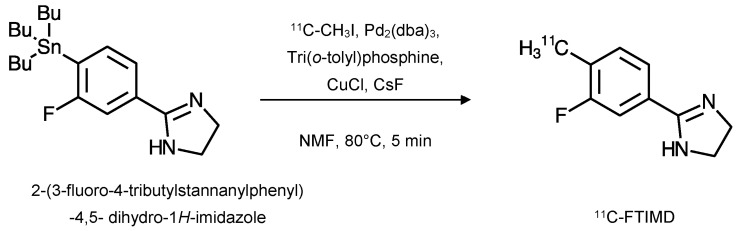
Synthesis of Ultra-High Specific Activity (UHSA) ^11^C-FTIMD. Tributylstannyl precursor (2-(3-fluoro-4-tributylstannanylphenyl)-4,5- dihydro-1*H*-imidazole) was mixed with a solution of tris(dibenzylideneacetone)dipalladium(0) (Pd_2_(dba)_3_; 1.4 μmol), tri(*o*-tolyl)phosphine (22 μmol), Copper(1) chloride (2.7 μmol), and cesium fluoride (7 μmol) in anhydrous *N*-methyl-2-pyrolidine (NMP) with ^11^C-methyl iodide. The reaction mixture was heated to 80 °C for 5 min. Purification was via HPLC and fractions of ^11^C-FTIMD were collected, dried, and redissolved in physiological saline for use (adapted from [2]).

An Ultra-High Specific Activity (UHSA) synthesis was also achieved for ^11^C-FTIMD (Figure 5). As with the normal specific activity synthesis, a palladium-promoted cross-coupling reaction was used, but with different conditions to those implemented previously. The carbon-11 was introduced using ^11^C-methyl iodide in the presence of tris(dibenzylideneacetone)dipalladium(0) (Pd_2_(dba)_3_; 1.4 μmol), tri(*o*-tolyl)phosphine (22 μmol), but with the addition of copper(1) chloride (2.7 μmol) and cesium fluoride (7 μmol) in anhydrous *N*-methyl-2-pyrolidine (NMP), rather than DMF. This had a lower radiochemical yield than the previous route (4.2 ± 0.7%; decay-corrected). However, it did produce a higher specific activity of 4470 ± 1660 GBq·µmol^−1^ at the end of the synthesis and a very high radiochemical purity of >99% [2].

#### 2.3.2. In Vitro Pharmacology

FTIMD, or compound 31 (2-(3′-Fluoro-4′-methylphenyl)-4,5-dihydro-1*H*-imidazole), was developed as part of a series of compounds for the imidazoline sites (I_1_ and I_2_) and adrenoceptor (α_1_ and α_2_). It showed a nanomolar affinity for the I_2_BS and K_i_ = 2.95 nM, with no apparent affinity at any of the other receptors tested (>10,000 nM) [16].

#### 2.3.3. In Vivo Preclinical

^11^C-FTIMD, from both the normal SA and UHSA, was tested in a range of preclinical animal species. The original SA synthesis of ^11^C-FTIMD was initially tested in both rats [3] and NHPs [6]. In both instances, it was found to show a specific signal and biodistribution consistent with brain regions rich in the I_2_BS. The uptake was able to be blocked via pre-treatment with a range of I_2_BS tracers and unlabelled FTIMD in rats [3] and BU224 (5 mg·kg^−1^) in NHPs [6]. An apparent low specific signal was observed in both species, with a 39% to 53% block due to BU224. The high affinity of FTIMD, ~3 nM, suggests that there is the potential for self-block, which could explain the low specific signal seen. To increase the specific signal, a UHSA synthesis of ^11^C-FTIMD was developed [2]. As with the normal SA ^11^C-FTIMD, the UHSA ^11^C-FTIMD showed a comparable specific signal and a biodistribution consistent with brain regions rich in the I_2_BS, which was able to be blocked with pre-treatment using BU224 (1 mg·kg^−1^) in rats. The specific signal achieved with the UHSA ^11^C-FTIMD was greater than that achieved using ^11^C-FTIMD with a normal specific activity (17–34% decrease) in all the brain regions investigated [2].

### 2.4. ^11^C-Metrazoline and ^11^C-TEIMD

#### 2.4.1. Radiochemistry

**Figure 6:**
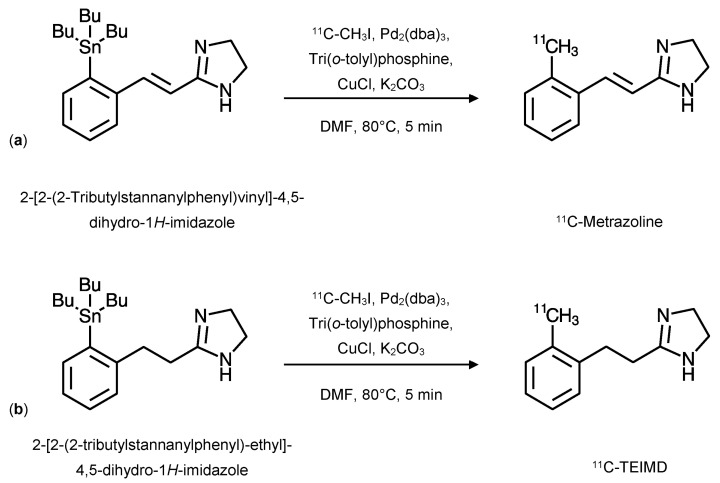
Synthesis of ^11^C-Metrazoline (**a**), and ^11^C-TEIMD (**b**). The synthesis of both radiotracers was performed using the same conditions, tributylstannyl precursors were mixed with a solution of tris(dibenzylideneacetone)dipalladium(0) (Pd_2_(dba)_3_; 3 μmol), tri(*o*-tolyl)phosphine (12 μmol), Copper(1) chloride (12 μmol), and potassium carbonate (12 μmol) in anhydrous *N*,*N*-dimethylformamide (DMF) with ^11^C-methyl iodide. The reaction mixture was heated to 80 °C for 5 min. Purification was via HPLC and fractions of the radiotracer were collected, dried, and redissolved in physiological saline for use (adapted from [23]).

Both ^11^C-Metrazoline and ^11^C-TEIMD were synthesised using a palladium-promoted cross-coupling reaction like that used for the ^11^C-FTIMD from the respective tributylstannyl precursor; 2-[2-(2-Tributylstannanylphenyl)vinyl]-4,5-dihydro-1*H*-imidazole for ^11^C-Metrazoline and 2-[2-(2-tributylstannanylphenyl)-ethyl]-4,5-dihydro-1*H*-imidazole for ^11^C-TEIMD. The reaction conditions were identical for both radiotracers (Figure 6). Both syntheses resulted in low radiochemical yields of 9.9% ± 4.9% and 13.6% ± 4.4% for ^11^C-metrazoline and ^11^C-TEIMD, respectively. The specific activities for both syntheses were 79 ± 40 GBq·µmol^−1^, with a radiochemical purity of >95% [23].

#### 2.4.2. In Vitro Pharmacology

Metrazoline, or compound 5 (2-[2-(*o*-tolyl)vinyl]-4,5-dihydro-1*H*-imidazole), was developed as a series of tracizoline analogues to develop 2D and 3D QSAR models for their binding to I_2_BS. It was found to have a sub-nanomolar affinity for the I_2_BS, K_i_ = 0.37 nM, and a low affinity for the α_2_adrenoceptor, K_i_ = 910 nM [17]. TEIMD, or compound 9 (2-[2-(*o*-tolyl)ethyl]-4,5-dihydro-1*H*-imidazole), was one of the two lead compounds synthesised to investigate the modulation of morphine analgesia seen with I_2_BS ligands. TEMID was based on the I_2_BS ligand, diphenyzoline, and showed the highest affinity for the I_2_BS (K_i_ = 1.7 nM) and a good selectivity over the I_1_ receptor (IC_50_ = 7360 nM) and α_2-_adrenoceptor (K_i_ = 557.7 nM) [18]. These pharmacological profiles made both these compounds attractive as potential PET tracers for the I_2_BS, which were developed by [23].

#### 2.4.3. In Vivo Preclinical

Both ^11^C-metrazoline and ^11^C-TEIMD were tested with small animal PETs using male mice and compared with ^11^C-FTIMD. Both these tracers showed peripheral uptake in several organs, as did ^11^C-FTIMD. This uptake was displaceable for ^11^C-metrazoline and ^11^C-FTIMD in the liver via BU224 (1 mg·kg^−1^). However, the uptake of ^11^C-TEIMD was not displaceable, indicating the lack of a specific signal. Furthermore, both ^11^C-metrazoline and ^11^C-TEIMD had very little to no brain uptake, respectively, indicating that they are both not as good I_2_BS PET tracers as ^11^C-FTIMD. However, ^11^C-metrazoline potentially has utility as a peripheral I_2_BS PET tracer [23].

## 3. Clinical Implementation of ^11^C-BU99008

### 3.1. Healthy Volunteers

**Figure 7:**
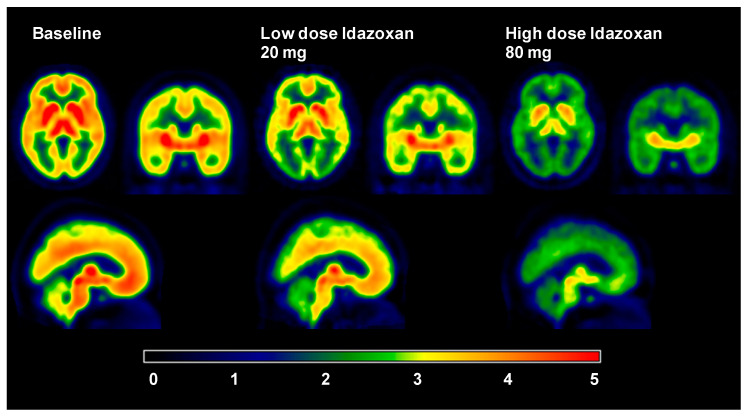
SUV images for healthy volunteers which demonstrates the heterogeneous brain uptake in all regions and dose-dependent blockade by idazoxan (p.o.) of ^11^C-BU99008 (Taken from [9]).

Of all the I_2_BS PET tracers developed, only one has progressed to being used in humans: ^11^C-BU99008. Its initial use was in healthy volunteers (HV) to assess its distribution, pharmacology, and kinetics, in order to determine its suitability for use in humans [9]. The uptake of ^11^C-BU99008 was found to be rapid and reversible. The metabolism data for ^11^C-BU99008 indicated that 10% of the parent radiotracer remained at 120 min. Arterial sampling was used to determine a plasma input function and thus enable the modelling of the time activity curves (TAC) and volumes of distribution (*V*_T_). Unlike in the pig [5] and rhesus [8], where a 1TCM and the MA1 models were found to best fit the kinetics of ^11^C-BU99008, respectively, it was found that a 2TCM best described the uptake of ^11^CBU99008 in humans [9]. The CNS distribution of ^11^C-BU99008 was heterogeneous and consistent with the known distribution of the I_2_BS and the distribution of ^11^C-BU99008 uptake/binding in preclinical species [5,8,15]. The *V*_T_ estimates were high in the striatum (105 ± 21 mL·cm^−3^), moderate in the cingulate cortex (62 ± 10 mL·cm^−3^), and low in the cerebellum (41 ± 7 mL·cm^−3^) [9]. The selectivity of ^11^C-BU99008 for the I_2_BS was determined using a heterologous competition. The subjects were pre-treated with different doses of the mixed I_2_BS/α_2_-adrenoceptor drug Idazoxan (IDX). IDX was used since it was the best clinically available I_2_BS ligand. The uptake of ^11^C-BU99008 was dose-dependently reduced throughout the brain, with an average block across all regions of about 60% (*V*_T,_ ~30 mL·cm^−3^) at the highest dose (80 mg) (Figure 7). The median effective dose for idazoxan was 28 mg. As with the NHP study using ^11^C-BU99008 [8], the role of MAO was investigated in the signal profile of ^11^C-BU99008 in humans. The subjects were pre-treated with an effective dose of the mixed MAO_A&B_ inhibitor isocarboxazid (50 mg). This pre-treatment did not alter the uptake of ^11^C-BU99008 in the CNS, further supporting the hypothesis that the I_2_BS and MAO are separate molecular entities. The test–retest reliability of ^11^C-BU99008 was also found to be within an acceptable range [9]. The shortcoming of ^11^C-BU99008 is that there appears to be no CNS reference region, meaning that arterial sampling is currently the only option for modelling this tracer. These data indicate that ^11^C-BU99008 is a good tracer for the study of the I_2_BS and its role in CNS disorders in vivo in humans.

### 3.2. Parkinson’s Disease

The I_2_BS has been reported to be associated with astrocytes [24], and so ^11^C-BU99008 has been categorised as an astrocytic tracer. As such, ^11^C-BU99008 has been utilised in several studies on neurodegenerative disorders, where glial perturbations are thought to be part of the pathophysiology of disorders such as Alzheimer’s disease (AD; see later section) and Parkinson’s disease (PD) [14]. In this study, 22 PD patients and 14 HVs were scanned with ^11^C-BU99008. The PD patients were sub-classified as those with early (*n* = 8) and moderate/advanced (*n* = 14) stages of the disease. In the early PD patients, there was an increase in the *V*_T_ of ^11^C-BU99008 across most of the brain regions reported, with many of these reaching significance. Many of the regions that showed this increase in ^11^C-BU99008 *V*_T_ were cortical, while only one of the sub-cortical regions, the brainstem, showed an increase in ^11^C-BU99008 *V*_T_, which also reached significance. Conversely, there was a significant reduction in ^11^C-BU99008 *V*_T_ in many brain regions reported for the moderate/advanced PD group compared with the HV and early PD groups. This loss in ^11^C-BU99008 *V*_T_ in the moderate/advanced PD group showed a negative correlation with disease duration and higher disease burden scores, which were measured with the Movement Disorder Society Unified Parkinson’s Disease Rating Scale (MDS-UPDRS), both of which were significant. In the moderate/advanced PD group, there was a significant positive correlation between the loss in ^11^C-BU99008 *V*_T_ and worse global cognitive scores, as assessed with the Montreal Cognitive Assessment in the frontal, temporal, and parietal cortices. To account for the loss of brain matter seen in PD, these data were subjected to a partial volume correction to correct for this. The conclusion drawn by the authors of this study was the importance of astrocytes in the initiation and progression of PD. The increase in ^11^C-BU99008 *V*_T_ in the early PD group was felt to reflect the neuroprotective compensatory mechanisms and pro-inflammatory upregulation of astrocytes. This increased astrocyte signal was lost as the disease progressed. This loss of astrocytes and the protection they provided in the cortex may have clinical relevance in the development of cognitive impairment [14].

A second study assessed the use of ^11^C-BU99008 in a different cohort of PD patients. These patients were diagnosed with Parkinson’s disease dementia (PDD) [13]. As with the previous study [14], this group used ^11^C-BU99008 as a marker to assess the role of astrocytes in the disease in comparison with HVs. The ^11^C-BU99008 or astrocytic “signal” measured in this study showed no significant difference between either the patient group or the healthy control group in any of the regions reported. Further subdivision of the healthy group, in order to provide closely age-matched controls, older healthy controls (OHC), and younger healthy controls (YHC), did not yield any change in these findings. However, this study did report a significant age-by-region interaction in the HVs, revealing an effect of age on the ^11^C-BU99008 signal. Evidence from post-mortem tissue studies confirms that the density of the I_2_BS varies with age in healthy individuals [25]. These data strongly suggest that the baseline measure of ^11^C-BU99008 may need to be normalised for age across subjects within a cohort, in order to allow for inter-subject comparison. The patient population in this study [13] was more comparable with the moderate/advanced Parkinson’s disease group from Wilson et al. [14], as this group showed increased cognitive decline, as shown by their scores on the MMSE and MoCA cognitive subscales.

Both PD studies discussed above showed an interesting relationship between the ^11^C-BU99008 (or astrocytic) signal in the potential progression of PD and in PD further complicated with significant dementia. The fact that the apparent loss of signal in moderate/advanced PD [14] was lost or returned to that of the age-matched controls in a similar population of PD with the added complication of dementia [13] is perplexing and clearly requires further investigation. The limitations of some of the sample sizes in these studies’ populations may provide some explanation, but it is unlikely to provide a complete explanation. It is more likely that these studies highlighted a very complex relationship of the ^11^CBU99008 signal, and by extension, astrogliosis in PD and PDD.

### 3.3. Alzheimer’s Disease

^11^C-BU99008 has also been used to investigate the role of astrocytes in other neurodegenerative disorders such as Alzheimer’s disease (AD), or more specifically, subjects with late-life cognitive impairment (CI) of the Alzheimer-type [11]. Eleven subjects with CI who were clinically diagnosed as having probable AD dementia or mild cognitive impairment (MCI) due to AD underwent ^11^C-BU99008 and ^18^F-florbetaben (tracer for amyloid (Aβ) deposition) PET scans, and these were compared with nine age-matched HVs. A region of interest (ROI) analysis showed a significant increase in the ^11^C-BU99008 *V*_T_ in the frontal cortexes of the AD patients compared with those of the HVs (17%, *p* = 0.007, uncorrected, two-tailed Student’s *t* test). A post hoc stratification of the CI group due to their Aβ status revealed that 8 of the 11 CI patients were Aβ+, while 3 were Aβ-. A further ROI analysis demonstrated an increase in ^11^C-BU99008 *V*_T_ in the Aβ+ CI subjects compared with the HVs in terms of the frontal (21%, *p* = 0.004), temporal (15%, *p* = 0.034), medial temporal (18%, *p* = 0.015), and occipital (24%, *p* = 0.026) lobes. To give a more detailed indication to the pattern of the distribution of the two PET tracers, voxel wise statistical parametric mapping (SPM) was performed. This analysis showed that, in the CI group and those who were Aβ+, an increased ^11^C-BU99008 uptake was demonstrated in the clusters of voxels predominantly in the cerebellum and the frontal, parietal, occipital, and temporal cortexes in comparison with the HVs, which was in broad agreement with the ROI analysis. This SPM analysis was further used to create a voxel-based biological parametric mapping (BPM) correlation analysis. Here, Z-score maps of the two PET tracers were compared with each other, demonstrating a strong positive correlation between the cortical ^11^C-BU99008 and ^18^F-florbetaben signals in the human cortexes of AD and Aβ+ subjects. To better interpret these data, this manuscript also reported in vitro autoradiographical and immunohistochemical experiments in the post-mortem human cortexes of AD and HV subjects using ^3^H-BU99008. These data showed that there was an increase in the ^3^H-BU99008 binding in the AD sections in comparison with the HV sections, which agreed with the in vivo ^11^C-BU99008 PET data. Immunostaining demonstrated a good spatial overlap of glial fibrillary acidic protein (GFAP; a widely recognised specific astrocytic marker) staining with that of higher ^3^H-BU99008 binding in adjacent sections, providing further evidence for the link between astrocytes and the I_2_BS. Another important observation from this manuscript was the inability of the unlabelled Aβ ligands, florbetaben, and PiB to displace any of the ^3^H-BU99008 binding. These data suggest that the ^3^H-BU99008 binding (I_2_BS), which was colocalised with both Aβ depositions and GFAP, was likely to be solely associated with astrocytes, with no contribution from the Aβ. These in vitro data, along with the data from the in vivo PET, indicated that the ^11^C-BU99008 PET signal was most likely to be associated with direct binding to the astrocytes, rather than directly to any Aβ depositions. This led the authors to suggest that ^11^C-BU99008 may be a useful tool for investigating the potential mechanistic link between Aβ deposition and astrocyte reactivity in neuropathology [11].

To further test this theory, the same group performed a deeper analysis of these data, but also included more detailed measures of metabolism and atrophy: glucose metabolism using ^18^F-FDG and grey matter (GM) atrophy using structural MRIs [12]. As expected, the whole brain ^18^F-FDG uptake was found to be significantly decreased in the Aβ+ CI patients compared with the HVs (*p* = 0.021). A further analysis showed that this global decrease in the Aβ+ CI patients was also significantly decreased compared with some specific brain regions in the HVs: frontal (*p* = 0.009), temporal (*p* < 0.001), medial temporal (*p* = 0.018), parietal (*p* < 0.001), and occipital (*p* < 0.001) lobes, as well as the posterior cingulate (*p* < 0.001) and hippocampus (*p* < 0.018). A structural analysis showed that the Aβ+ CI patients had a significantly globally reduced GM volume (*p* = 0.046), which also reached significance in several brain regions: temporal (*p* = 0.029), medial temporal (*p* = 0.009), and parietal (*p* = 0.020) lobes, as well as the posterior cingulate (*p* = 0.036) and hippocampus (*p* = 0.032). In addition, a further comparison of the AD versus MCI patients (irrespective of Aβ status) showed a significant increase in the ^11^C-BU99008 uptake in the MCI patients in the anterior (*p* = 0.023) and posterior (*p* = 0.007) cingulates [12]. With these data [11,12] and evidence from other published studies, the authors hypothesised a detailed role for astrocytes in AD progression in a normal healthy aging brain, through MCI due to AD to “full” AD. This model suggests that astrocytic activity increases in the early, pre-symptomatic stages of AD, peaking in the MCI/early AD stages and decreasing in the advanced stages of AD [12]. The implication of this is that increased astrocyte activity could be an early indicator of AD pathology and, in turn, an astrocytic marker such as the I_2_BS tracer ^11^C-BU99008 could be valuable for a better understanding of the development and progression of AD, or even act as a diagnostic aid.

Recently, Kumar et al. [26] performed an elegant series of in vitro studies that compared the tritiated and “cold” versions of BU99008 with (to date) the only other validated astrocytic PET tracer, ^11^C-deutrium-l-deprenyl (^11^C-DED) [27]. ^11^C-DED targets the monoamine oxidase B enzyme (MAO_B_) [28] rather than the I_2_BS. These studies showed that both of these astrocytic markers were highly comparable, providing further evidence that ^11^C-BU99008 targets astrocytes. In addition, this same group published a review on the role of astrogliosis in AD that further posited a temporal variation in astrogliosis throughout the progression of AD, which is in keeping with the results reported in the above studies (see [29]).

## 4. Discussion

Acknowledging the current limited level of clinical PET data for I_2_BS PET tracers, these reports represent an important early view of how the role of astrogliosis may impact early decline in both AD and PD, and specifically, if there is a mechanistic link between Aβ deposition and astrocyte reactivity in the neuropathology of disease. From a drug development perspective, understanding the role of astrocytes in CNS disorders could be of vital importance for comprehending their function in neurodegenerative disease. In particular, if astrogliosis is proven to precede the activation of microglia in neurodegenerative disorders, then this could be an area of focus and of particular value for probing the early stages of neurodegeneration; hence, this may represent a novel means of detecting detrimental changes earlier in the disease process than previously possible. Consequently, the development of novel therapeutic interventions that could look to target astrogliosis, either as complementary to microglia or standalone, may need to be a future consideration in researching neurodegeneration.

These clinical reports also highlighted the need for a clear definition of a harmonised set of criteria characterising “early” versus “late” disease in both the AD and PD settings, which has emerged as a crucial finding from these reports. This is especially true if astrogliosis is deemed to be of importance in determining the chronology of the disease process and would also allow for a synchronisation of the data across sites from future studies.

Targeting neuroinflammation, and in particular microglial activation, is a current area of the focus of new treatment interventions for a number of neurodegenerative disorders. Historically, imaging neurodegeneration with TSPO PET tracers was implicated with imaging the microglial response. TSPO PET tracers have evolved from the first-generation tracer (^11^C-PK11195) through to a host of clinically available second-generation tracers (e.g., ^11^C-PBR28 and ^18^F-DPA714), with the use of second-generation TSPO PET tracers limited by the clinical variability displayed among individuals in relation to the TSPO binding potential (BP). The cause of this variability was shown to be due to a single nucleotide polymorphism (rs6971) influencing the binding affinity of the TSPO tracers, thereby leading to three distinct binding statuses: high-, mixed-, and low-affinity binders (HAB, MAB, and LAB, respectively). More recently, third-generation TSPO PET tracers (^11^C-ER176 and ^18^F-GE-180) have been developed with a view to overcoming the challenge of the single nucleotide polymorphism, in order to enable these tracers to bind equally to the TSPO in all subjects clinically [30]. The data published recently however, have shown TSPO to be expressed in multiple cell types, including microglia, astrocytes, neurones, and endothelial cells [31]. With the development of more focused, clinically available PET imaging tracers for the microglia, including those that target the P2X7 (purported to be mainly expressed in microglia and astrocytes; [32]) and CSF1R (purported to be mainly expressed in microglia; [33]), there now exists the opportunity to probe the microglia more exclusively, along with their roles in neurodegenerative disease. Combining these clinical imaging tools that probe both the microglia and astrocytes, i.e., CSF1R (mainly microglia), I_2_BS (mainly astrocytes), and P2X7 (both microglia and astrocytes), should allow for a more detailed examination of the roles that these two cell types play in neurodegeneration, along with any inter-dependencies. Operationally, the clinical implementation of (in terms of GMP validation) and ease of access to these tracers (in terms of proprietary use) still needs to be fully considered to enable larger studies to occur at sites near relevant patient populations. Probing and understanding the relevance of the chronology of astrogliosis in relation to the activated microglia in neurodegenerative disease is still in its infancy, and will only be more fully understood and potentially validated with the use of I_2_BS PETs in additional studies on neurodegenerative disease.

## 5. Summary

To summarise, this review describes the current status of I_2_BS imaging. However, the likelihood that the I_2_BS is a marker for astrocytes has meant that much of the utility of these tracers, especially clinically, has been in probing how these cells are involved in neurodegeneration. Five compounds have been radiolabelled and evaluated as potential PET tracers, with only one (^11^C-BU99008) currently validated to GMP for clinical use. Clinical studies utilising ^11^C-BU99008 have revealed the potential early involvement of astrogliosis in neurodegeneration that might precede the activation of microglia, which, if confirmed, provides a vital new route for potentially targeting neurodegeneration earlier in the disease course.

## Figures and Tables

**Table 1:** Summary of the five PET tracers developed for the I_2_BS.

Name	K_i_ (nM)	CNS I2 Binding Specificity	CNS Entry	In Vivo PET (Species)
^11^C-FTIMD	2.95 ^†^	Low (18–34% rat; 25–34% NHP specific signal) [3,6]	High	Rat, NHP
^11^C-Metrazoline	0.37 ^‡^	Low	Low	Mice
^11^C-TEIMD	1.7 ^^^	Low	Low	Mice
^18^F-FEBU (^18^F-BU99018)	2.6 ^#^	Moderate (~75% specific signal) [4]	High	Mice, Rat
^11^C-BU99008	1.4 ^#^	High [8,15]	High	Rat, Pig, NHP, Human

Equilibrium inhibitory constant (K_i_) values and species/tissue source taken from: ^†^—rabbit kidney cortex [16]; ^‡^—rabbit kidney cortex [17]; ^^^—rat brain [18]; and ^#^—rat whole brain [15].
